# Identification of Tumor Necrosis Factor-Alpha (TNF-α) Inhibitor in Rheumatoid Arthritis Using Network Pharmacology and Molecular Docking

**DOI:** 10.3389/fphar.2021.690118

**Published:** 2021-05-21

**Authors:** Liang Liang Bai, Hao Chen, Peng Zhou, Jun Yu

**Affiliations:** ^1^ School of Biomedical Engineering, Anhui Medical University, Hefei, China; ^2^ School of Integrated Chinese and Western Medicine, Anhui University of Chinese Medicine, Institute of Integrated Chinese and Western Medicine, Anhui Academy of Chinese Medicine, Anhui Province Key Laboratory of Chinese Medicinal Formula, Hefei, China; ^3^ The Fourth Affiliated Hospital, Anhui Medical University, Hefei, China

**Keywords:** immune inflammatory, rheumatoid arthritis, *Paeonia lactiflora* Pallas, network pharmacology, molecular docking, tumor necrosis factor-alpha inhibitor

## Abstract

**Background:** This study aimed to investigate the molecular mechanism of *Radix* Paeoniae Alba (white peony, WP) in treating immune inflammatory diseases of rheumatoid arthritis (RA) and tumor necrosis factor-alpha (TNF-α) inhibitors (TNFis) by using network pharmacology and molecular docking.

**Methods:** In this study, the ingredient of WP and the potential inflammatory targets of RA were obtained from the Traditional Chinese Medicine Systematic Pharmacology Database, GeneCard, and OMIM databases, respectively. The establishment of the RA–WP-potential inflammatory target gene interaction network was accomplished using the STRING database. Network maps of the WP–RA-potential inflammatory target gene network were constructed using Cytoscape software. Gene ontology (GO) and the biological pathway (KEGG) enrichment analyses were used to further explore the RA mechanism and therapeutic effects of WP. Molecular docking technology was used to analyze the optimal effective components from WP for docking with TNF-α.

**Results:** Thirteen active ingredients and 71 target genes were screened from WP, and 49 of the target genes intersected with RA target inflammatory genes and were considered potential therapeutic targets. Network pharmacological analysis showed that the WP active ingredients such as mairin, DPHCD, (+)-catechin, beta-sitosterol, paeoniflorin, sitosterol, and kaempferol showed better correlation with RA inflammatory target genes such as PGR, PTGS1, PTGS2, NR3C2, TNFSF15, and CHRM2, respectively. The immune-inflammatory signaling pathways of the active ingredients for the treatment of RA are the TNF-α signaling pathway, Toll-like receptor signaling pathway, cell apoptosis, interleukin-17 signaling pathway, C-type lectin receptor signaling pathway, mitogen-associated protein kinase, *etc*. Molecular docking results suggested that mairin was the most appropriate natural TNFis.

**Conclusion:** Our findings provide an essential role and basis for further immune-inflammatory studies into the molecular mechanisms of WP and TNFis development in RA.

## Introduction

Rheumatoid arthritis (RA) is a severe inflammatory autoimmune disease that is associated with multiple joint disabilities, systemic complications, lower quality of life, and high mortality, and affects ∼1% of the population worldwide (Mcinnes and Schett, 2011; Chen et al., 2019). The main clinical features of RA are joints, such as the hands, wrists, and feet, showing redness, swelling, heat, and pain and body dysfunction, which may appear as erosive deformities in advanced-stage disease (Mikuls et al., 2016). Currently, steroidal anti-inflammatory drugs (glucocorticoids), nonsteroidal anti-inflammatory drugs, immunosuppressive drugs (chemical drugs and biological drugs), and Chinese natural medicines are applied to treat RA in the clinic (Bijlsma et al., 2015; Lampropoulos et al., 2015; Wei, 2016). However, the adverse effects of the medication, especially serious toxicity, such as the liver, kidney, gastrointestinal, respiratory, blood, cardiovascular, bone marrow, nerve, and malignant infection, can confuse RA patients (Zhang et al., 2014; Felson, 2016; Solomon et al., 2018). This is significant in terms of the development of natural products with therapeutic potential for clinical applications, which has attracted growing attention in recent years (Zhang and Wei, 2020).

*Paeonia lactiflora* Pallas is an herbaceous perennial flowering plant in the family Paeoniaceae with fleshy roots and annual stems (He and Dai, 2011). The dried root without bark of *Paeonia lactiflora* Pallas, namely, *Radix Paeoniae Alba*, has been used as a medicinal herb to treat pain, inflammation, and immune disorders for more than 1000 years in traditional Chinese medicine (TCM) (Zhang and Wei, 2020). The Chinese name for *Radix Paeoniae Alba* is white peony (WP), which possesses a variety of active constituents with anti-inflammatory, hepatic protective, analgesic, and immunoregulatory functions (He et al., 2010; Yan et al., 2018). A water/ethanol extract of WP is known as total glucosides of peony (TGP), which is reported to have a significant therapeutic effect on RA (Jia et al., 2014; Wang et al., 2015). In addition, TGP was approved by the National Medical Products Administration in 1998 as an anti-inflammatory and immunomodulatory drug and has been widely used in many hospitals in China (Min et al., 2005). Long-term clinical application has found that, except for gastrointestinal reactions, the use of TGP has no serious adverse reactions.

Tumor necrosis factor-alpha (TNF-α) is an inflammatory cytokine that mediates key roles in proliferation, differentiation, apoptosis, immune regulation, and inflammation induction (Wijbrandts et al., 2008). TNF-α dysfunction can cause many diseases, such as RA, psoriasis, and ankylosing spondylitis (Hyrich et al., 2007; Schulz et al., 2014). Due to the important role of TNF-α in the inflammatory process, the inhibition of its activity as a target for drug research has attracted growing attention in recent years. Drugs targeting TNF-α have been successfully used in the treatment of various inflammatory diseases, such as infliximab, etanercept, adalimumab, and golimumab (Jin et al., 2010; Inman et al., 2016). These TNF-α inhibitors (TNFis) have been approved by the Food and Drug Administration for the treatment of RA, Crohn’s disease, psoriasis, ankylosing spondylitis, *etc*. The annual global sales of TNFis have exceeded tens of billions of dollars, becoming one of the world’s best-selling drugs. However, RA patients receiving TNFis are more likely to discontinue therapy in the advanced years because of severe side effects and injection reactions (Alonso-Ruiz et al., 2008; Jin et al., 2010).

Our previous study found that in traditional Chinese medicine, WP and its main active ingredients have shown excellent anti-inflammatory effects, and its target of action is related to the inhibition of TNF-α. For example, the administration of TGP (25, 50, and 100 mg kg^−1^, ig 14 days) inhibits secondary inflammatory reactions and histological and synovial ultrastructural changes in collagen-induced arthritis (CIA) (CIA in rats is similar to human RA in both its clinical and histopathological features) and adjuvant arthritis (AA) in rats. In addition, the level of TNF-α produced by macrophage-like synoviocytes (MLSs) from CIA and AA rats was decreased by TGP administration (Zhu et al., 2005; Xu et al., 2007; Zhang et al., 2007). Furthermore, in an RA patient trial, TGP significantly inhibited fibroblast-like synoviocyte proliferation induced by TNF-α (Wu and Zeng, 2007). Based on WP and its main active ingredients having certain representativeness and wide application in anti-RA therapy, we used molecular docking technology to analyze the most suitable active ingredients of WP as natural TNFis for the development of new and effective TNFis, which is of great significance.

**FIGURE 1:**
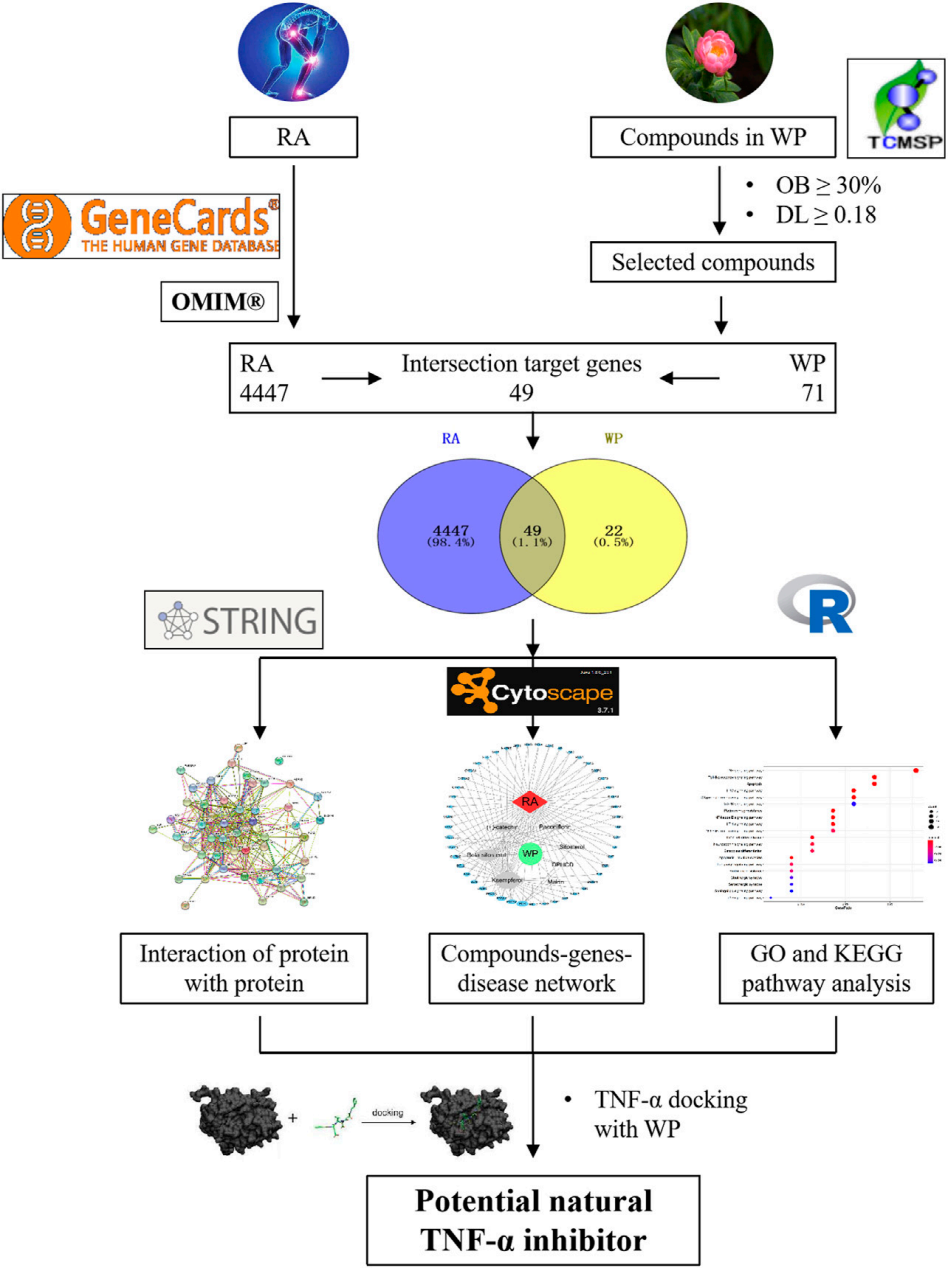
The workflow of TNF-α inhibitor prediction in RA.

Network pharmacology is a meaningful approach to drug discovery (Jiang et al., 2019). In this study, the effective ingredients of WP were first screened. Then, we analyzed and summarized the targets of the active ingredients in the treatment of RA. Finally, these targets were used to research the active ingredient’s targets and target pathways by using network pharmacology, gene ontology (GO), and biological pathway (KEGG) functional enrichment. Based on the results summarized above, molecular docking technology was used to analyze the optimal effective components from WP that dock with TNF-α to explore the most appropriate compound for the development of new and effective TNFis. This research was carried out to provide a theoretical basis for the molecular mechanisms of WP against RA. The workflow is shown in Figure 1.

## Materials and Methods

### WP Active Ingredient Database Establishment

The ingredients of WP were searched and collected from the Traditional Chinese Medicine Systems Pharmacology Database and Analysis Platform (TCMSP, https://tcmspw.com/index.php), which includes a network of chemicals, targets, and associated drug targets, as well as the pharmacokinetic properties of natural compounds such as oral bioavailability (OB), drug-likeness (DL), intestinal epithelial permeability, blood–brain barrier penetrability, and water solubility. Based on the collection of ingredients described above, 13 active ingredients were obtained with OB ≥ 30% and DL ≥ 0.18 as the screening conditions (Ru et al., 2014). For the preparation of molecular docking, 13 active ingredient sdf files were downloaded from the PubChem database (http://pubchem.ncbi.nlm.nih.gov) and saved as mol2 files after energy minimization calculations by Chemdraw 3D Ultra software.

### Clustering of WP- and RA-Related Target Genes

The WP-related target genes were clustered depending on chemical similarities and pharmacophore models *via* the TCMSP database. These WP-related target names were calibrated to the standardized name using the UniProt database (https://www.uniprot.org/; Szklarczyk et al., 2016).

RA-related target genes were collected from two databases, namely, GeneCard (https://www.genecards.org/) and OMIM (Online Mendelian Inheritance in Man, https://omim.org/search/advanced/geneMap) (Li et al., 2018). Potential target genes (i.e., overlapping target genes) of WP therapy for RA were acquired through the Veeny 2.1 (https://bioinfogp.cnb.csic.es/tools/venny/) intersection.

### Protein–Protein Interaction (PPI) Network Map of the WP–RA-Potential Target Genes

A PPI network map was constructed for the co-expression, fusion, neighborhood, and co-localization of potential target genes with predicted gene interactions (Qin et al., 2020). The name of the potential target genes was entered *via* the STRING database (https://string-db.org/) with “*Homo sapiens*” being selected. Each node represents a protein in the PPI network map, and each edge represents a functional association between potential target genes. These results were imported into Cytoscape and used for network production and analysis.

### Construction of the WP–RA-Potential Target Gene Network

A visual network was established through Cytoscape software (version 3.7.1) to reflect the complex relationship between WP and RA-potential target genes. Active ingredients and gene targets were obtained by Cytoscape analysis (Zuo et al., 2018). In the visual network, each node represents the compounds and target genes, while lines indicate the intermolecular interactions between compounds and target genes. The network topology parameters were analyzed to select the key compounds and target genes.

### GO and KEGG Pathway Enrichment Analysis

First, potential target gene names were transformed into entrezID by the R package “org.Hs.eg.db, version = 3.8,” which helps to exclude errors caused by capitalization or abbreviations of the target name. Then, GO biological functions and the KEGG pathway enrichment analysis were visualized with the R packages “DOSE,” “clusterProfiler,” and “pathview,” for which the *p*-value was <0.05 for further analysis.

### Binding Capacity Between Active Ingredients and Key Target Genes by Molecular Docking

Docking of active ingredients selected from the WP–RA-potential target gene network to the TNF-α receptor was explored using CB-Dock online molecular docking (http://cao.labshare.cn/cb-dock//) (Liu et al., 2020; Tao et al., 2020). PDF files for TNF-α (protein ID is 2az5) and the active ingredients (files prepared from “WP Active Ingredient Database Establishment” section) were uploaded to the CB-Dock website. After determining the docking pocket coordinates, molecular docking and conformational scoring were performed using CB-dock. The lower the vina scores are, the more stable is the ligand binding to the receptor, which was used for preliminary evaluation of the binding activity of the compound to the target.

## Results

### WP Active Ingredient Database Establishment

A total of 85 WP ingredients were searched and collected from the TCMSP. Based on OB ≥ 30% and DL ≥ 0.18 as the screening conditions, 13 active ingredients were selected from the WP ingredients for database establishment (Table 1). These active ingredients included 11alpha,12alpha-epoxy-3beta-23-dihydroxy-30-norolean-20-en-28,12beta-olide, paeoniflorgenone, (3S,5R,8R,9R,10S,14S)-3,17-dihydroxy-4,4,8,10,14-pentamethyl-2,3,5,6,7,-9-hexahydro-1H-cyclopenta [a]phenanthrene-15,16-dione (DPHCD), lactiflorin, paeoniflorin, paeoniflorin_qt, albiflorin_qt, benzoyl paeoniflorin, mairin, beta-sitosterol, sitosterol, kaempferol, and (+)-catechin.

**TABLE 1:** Characteristics of active ingredients in WP.

No	Molecule ID	Molecule name	Molecular weight	OB (%)	DL
1	MOL001910	11alpha,12alpha-epoxy-3beta-23-dihydroxy-30-norolean-20-en-28,12beta-olide	470.71	64.77	0.38
2	MOL001918	Paeoniflorgenone	318.35	87.59	0.37
3	MOL001919	DPHCD	358.52	43.56	0.53
4	MOL001921	Lactiflorin	462.49	49.12	0.8
5	MOL001924	Paeoniflorin	480.51	53.87	0.79
6	MOL001925	Paeoniflorin_qt	318.35	68.18	0.4
7	MOL001928	Albiflorin_qt	318.35	66.64	0.33
8	MOL001930	Benzoyl paeoniflorin	584.62	31.27	0.75
9	MOL000211	Mairin	456.78	55.38	0.78
10	MOL000358	Beta-sitosterol	414.79	36.91	0.75
11	MOL000359	Sitosterol	414.79	36.91	0.75
12	MOL000422	Kaempferol	286.25	41.88	0.24
13	MOL000492	(+)-Catechin	290.29	54.83	0.24

OB: oral bioavailability; DL: drug-likeness; DPHCD: (3S,5R,8R,9R,10S,14S)-3,17-dihydroxy-4,4,8,10,14-pentamethyl-2,3,5,6,7,9-hexahydro-1H-cyclopenta[a]phenanthrene-15,16-dione.

### Potential Target Genes and the PPI Network Map of WP Therapy for RA

**FIGURE 2:**
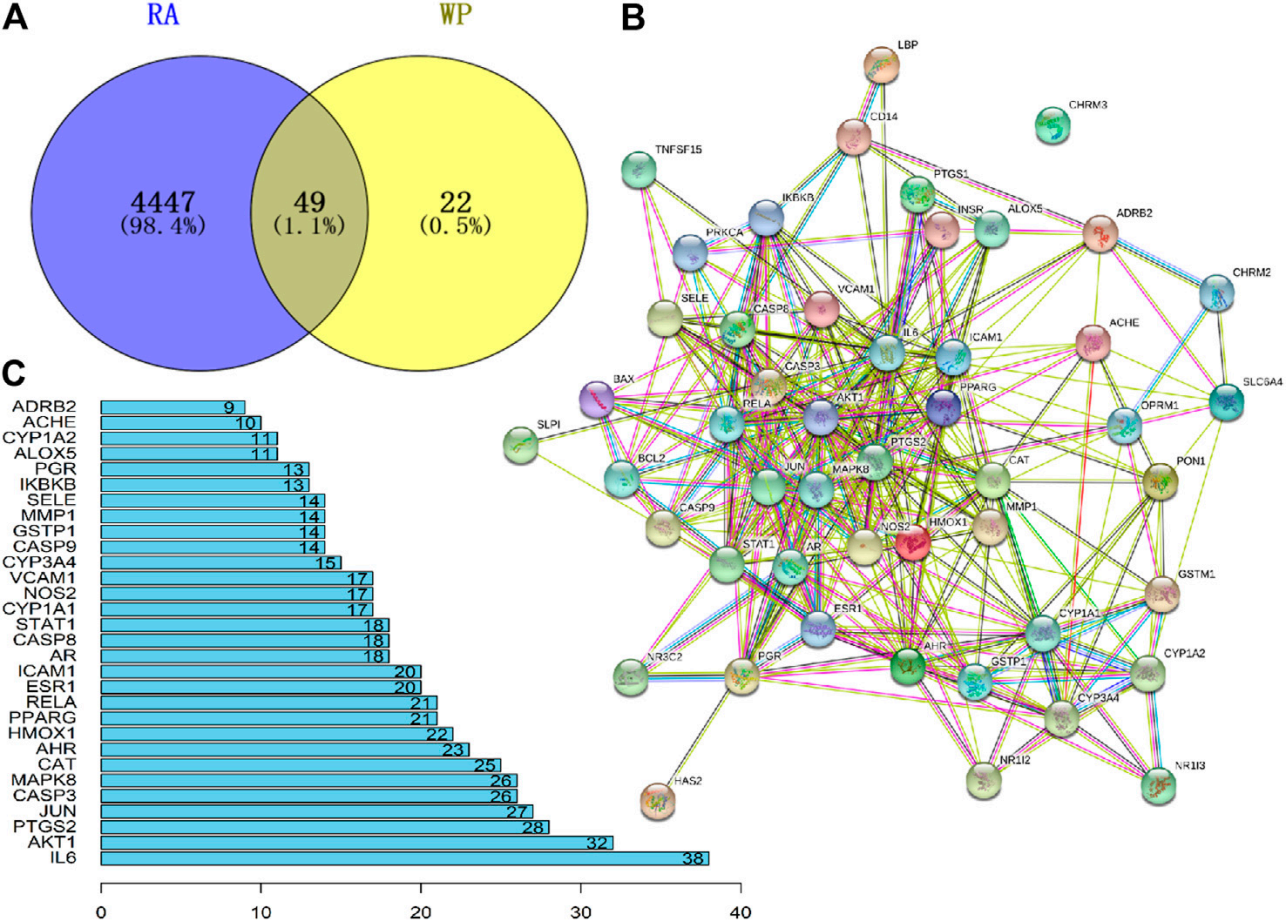
Potential target genes and PPI network map of WP therapy for RA. **(A)** The Venny results of potential target genes of WP therapy for RA. **(B)** The PPI network map of 49 target genes. **(C)** Count and list of the top 30 genes of PPI network map.

The GeneCard and OMIM databases were searched, yielding a total of 4,447 RA target genes, excluding duplicates. Similarly, 95 target genes were obtained from the TCMSP database for 13 active ingredients of WP (Supplementary Table S1). The removal of duplicates after verification yielded 71 target genes. RA target genes and WP target genes were intersected using Venny 2.1 software to obtain 49 potential targets (Figure 2A) (Table 2). Sequentially, the 49 target genes were imported into the STRING database to obtain the PPI network map (Figure 2B). The top 30 genes of the PPI network results were identified and listed by the R package “Venn Diagram” (Figure 2C). These genes include interleukin-6 (IL-6), RAC-alpha serine/threonine-protein kinase (AKT1), prostaglandin G/H synthase 2 (PTGS2), transcription factor AP-1 (JUN), caspase-3 (CASP3), and mitogen-activated protein kinase 8 (MAPK8).

**TABLE 2:** 49 potential target genes of WP therapy for RA.

No.	Target	Symbol	Entrez ID	No.	Target	Symbol	Entrez ID
1	Progesterone receptor	PGR	5,241	26	Transcription factor p65	RELA	5,970
2	Mineralocorticoid receptor	NR3C2	4,306	27	Inhibitor of nuclear factor kappa-B kinase subunit beta	IKBKB	3,551
3	Tumor necrosis factor	TNFSF15	9,966	28	RAC-alpha serine/threonine-protein kinase	AKT1	207
4	Interleukin-6	IL6	3,569	29	Mitogen-activated protein kinase 8	MAPK8	5,599
5	Monocyte differentiation antigen CD14	CD14	929	30	Interstitial collagenase	MMP1	4,312
6	Lipopolysaccharide-binding protein	LBP	3,929	31	Signal transducer and activator of transcription 1-alpha/beta	STAT1	6,772
7	Prostaglandin G/H synthase 1	PTGS1	5,742	32	Heme oxygenase 1	HMOX1	3,162
8	Prostaglandin G/H synthase 2	PTGS2	5,743	33	Cytochrome P450 3A4	CYP3A4	1,576
9	Muscarinic acetylcholine receptor M3	CHRM3	1,131	34	Cytochrome P450 1A2	CYP1A2	1,544
10	Muscarinic acetylcholine receptor M2	CHRM2	1,129	35	Cytochrome P450 1A1	CYP1A1	1,543
11	Beta-2 adrenergic receptor	ADRB2	154	36	Intercellular adhesion molecule 1	ICAM1	3,383
12	Sodium-dependent serotonin transporter	SLC6A4	6,532	37	E-selectin	SELE	6,401
13	Mu-type opioid receptor	OPRM1	4,988	38	Vascular cell adhesion protein 1	VCAM1	7,412
14	Apoptosis regulator Bcl-2	BCL2	596	39	Nuclear receptor subfamily 1 group I member 2	NR1I2	8,856
15	Apoptosis regulator BAX	BAX	581	40	Arachidonate 5-lipoxygenase	ALOX5	240
16	Caspase-9	CASP9	842	41	Hyaluronan synthase 2	HAS2	3,037
17	Transcription factor AP-1	JUN	3,725	42	Glutathione S-transferase *p*	GSTP1	2,950
18	Caspase-3	CASP3	836	43	Aryl hydrocarbon receptor	AHR	196
19	Caspase-8	CASP8	841	44	Nuclear receptor subfamily 1 group I member 3	NR1I3	9,970
20	Protein kinase C alpha type	PRKCA	5,578	45	Insulin receptor	INSR	3,643
21	Serum paraoxonase/arylesterase	PON1	5,444	46	Glutathione S-transferase Mu 1	GSTM1	2,944
22	Nitric oxide synthase, inducible	NOS2	4,843	47	Antileukoproteinase	SLPI	6,590
23	Androgen receptor	AR	367	48	Estrogen receptor	ESR1	2099
24	Peroxisome proliferator- activated receptor gamma	PPARG	5,468	49	Catalase	CAT	847
25	Acetylcholinesterase	ACHE	43				

### Construction and Analysis of the WP–RA-Potential Target Gene Network

**FIGURE 3:**
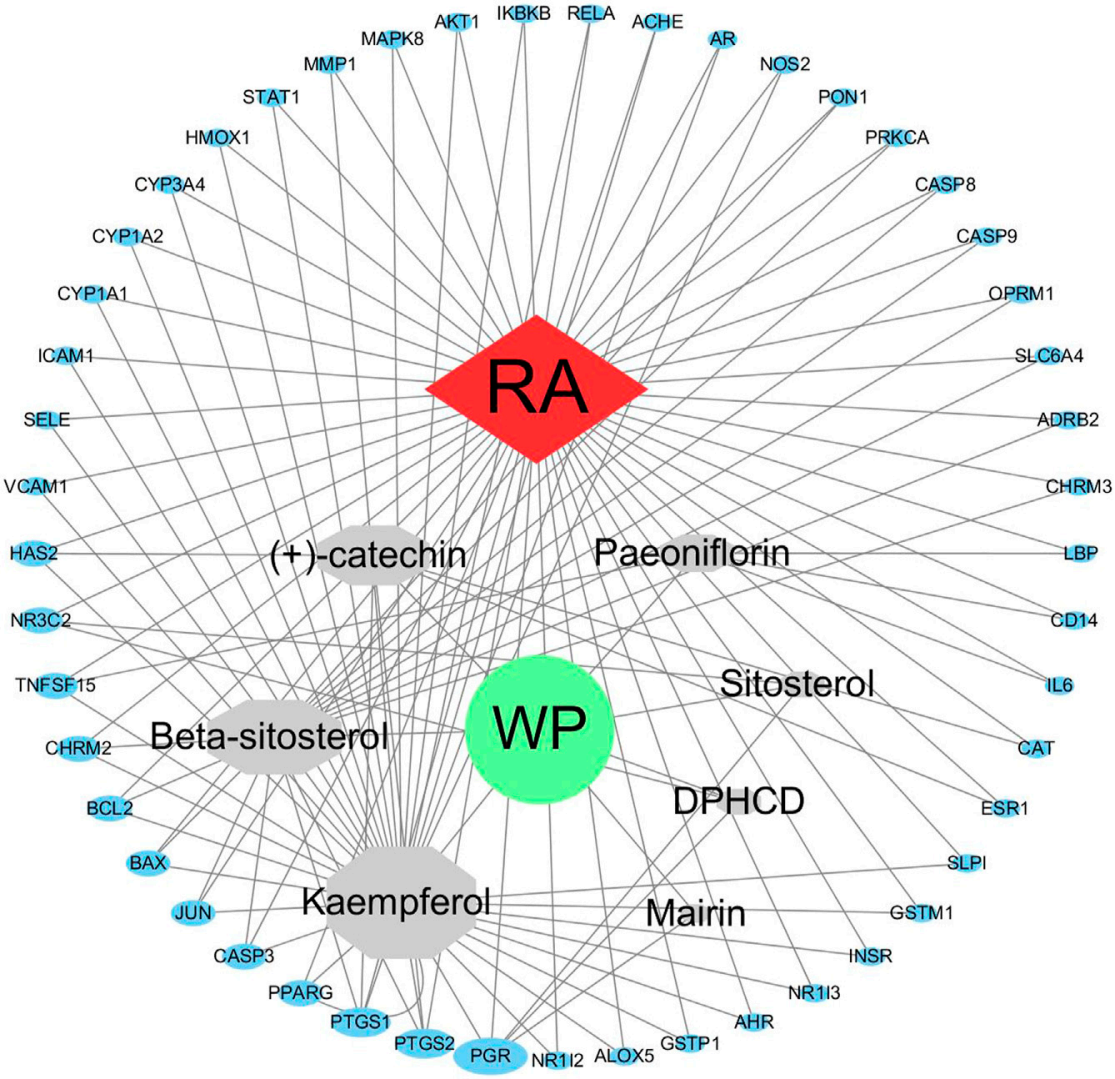
The WP–RA-potential target gene network. The size of each node in the network represents the size of its degree value. The gray connecting lines indicate that each node is interconnected.

We have to enter the results of Supplementary Table S1 and Table 2 into the Cytoscape software (version 3.7.1) to obtain a WP–RA-potential target gene network (Figure 3). A total of 58 nodes and 242 lines were obtained from the WP–RA-potential target gene network. Further analysis found that the top 12 targets, namely, progesterone receptor (PGR), PTGS1, PTGS2, mineralocorticoid receptor (NR3C2), TNFSF15, muscarinic acetylcholine receptor M2 (CHRM2), apoptosis regulator Bcl-2 (BCL2), apoptosis regulator BAX (BAX), JUN, CASP3, peroxisome proliferator–activated receptor gamma (PPARG), and hyaluronan synthase 2 (HAS2), have a higher degree in this process, which explains their significance in the network (degree >3) (Table 3).

**TABLE 3:** Top 12 high degree genes in the network.

No	Target	Symbol	Degree
1	Progesterone receptor	PGR	5
2	Prostaglandin G/H synthase 1	PTGS1	4
3	Prostaglandin G/H synthase 2	PTGS2	4
4	Mineralocorticoid receptor	NR3C2	3
5	Tumor necrosis factor	TNFSF15	3
6	Muscarinic acetylcholine receptor M2	CHRM2	3
7	Apoptosis regulator Bcl-2	BCL2	3
8	Apoptosis regulator BAX	BAX	3
9	Transcription factor AP-1	JUN	3
10	Caspase-3	CASP3	3
11	Peroxisome proliferator-activated receptor gamma	PPARG	3
12	Hyaluronan synthase 2	HAS2	3

### GO and KEGG Pathway Enrichment Analysis

**FIGURE 4:**
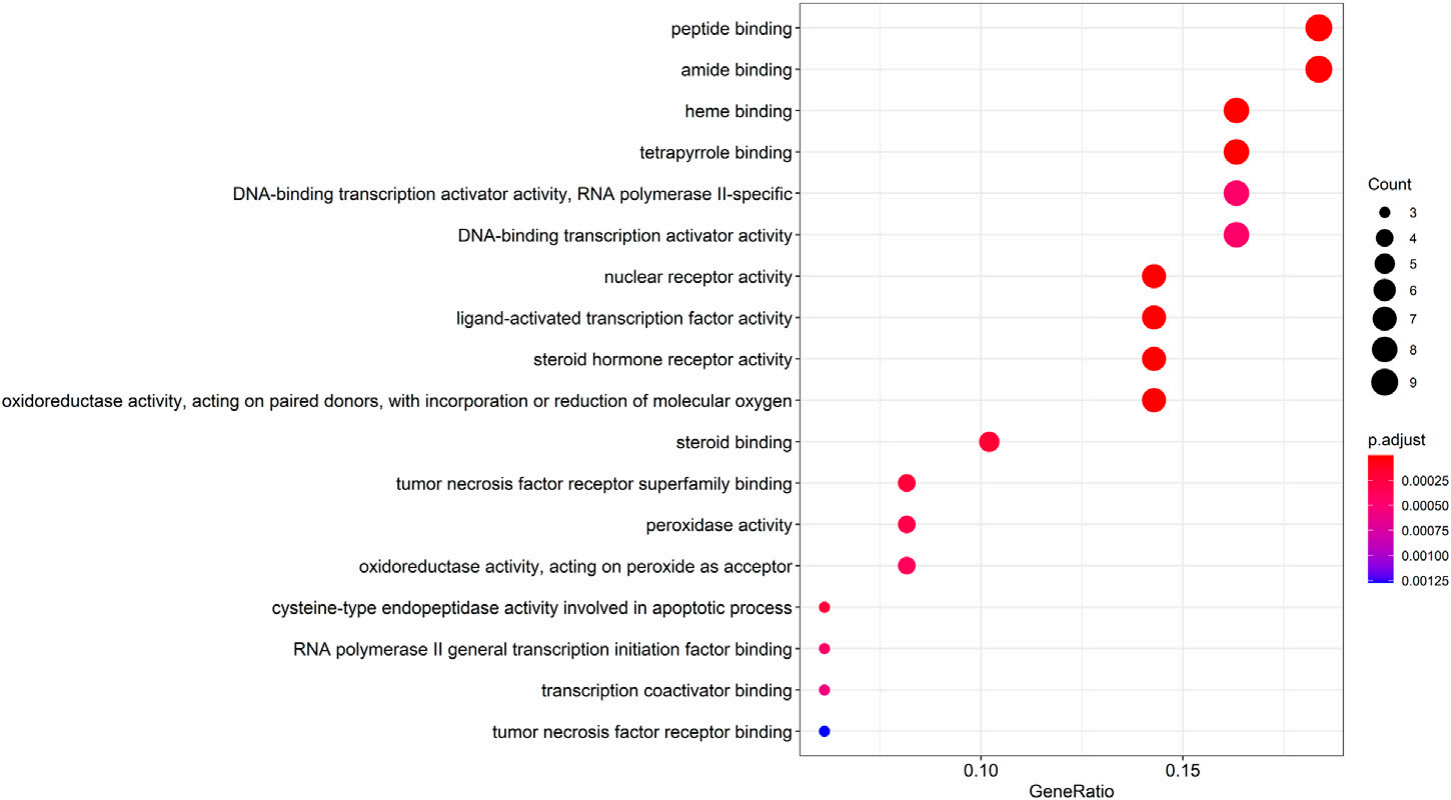
The top 18 remarkably enriched GO analysis for biological function of potential target genes of WP in RA.

The entrezIDs of 49 target genes are shown in Table 2. A total of 79 genes’ biological functions were obtained from GO enrichment analysis (*p* < 0.05). The top 18 markedly enriched gene biological function catalogs were selected for the generation of scatterplots (Figure 4) and histograms (Supplementary Figure S1). The biological functions of these genes mainly include peptide binding, amide binding, heme binding, tetrapyrrole binding, and DNA-binding transcription activator activity*.* These results show that WP is involved in the treatment of RA through a variety of gene biological functions that contribute to the understanding of the anti-inflammatory and immune-modulatory mechanisms in RA.

**FIGURE 5:**
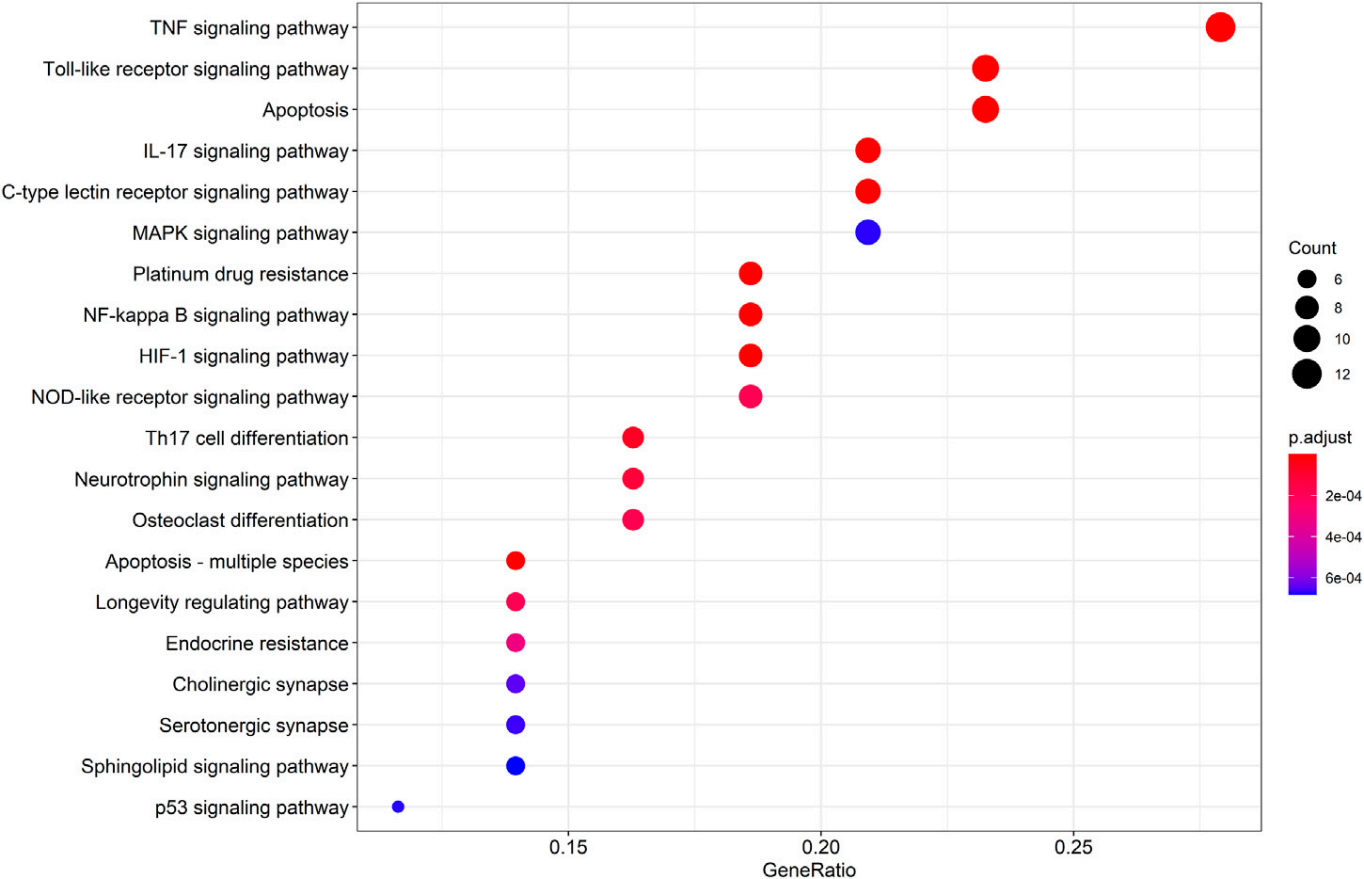
The top 20 remarkably enriched KEGG analysis for the signaling pathway of potential target genes of WP in RA.

**FIGURE 6:**
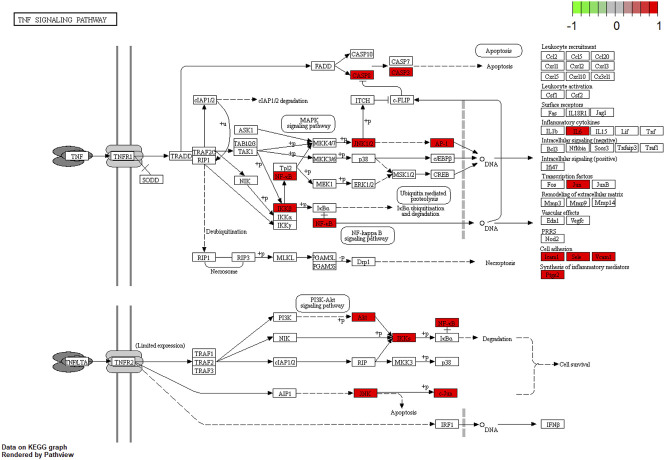
The TNF-α signaling pathway of potential target genes of WP in RA. Arrows indicate upstream and downstream relationships between genes. The red is a WP target gene in network.

To gain a profound understanding of the mechanism of action of WP in the treatment of RA, a total of 50 signaling pathways were obtained from the KEGG pathway enrichment analysis (*p* < 0.05). Scatterplots (Figure 5) and histograms (Supplementary Figure S2) are shown by using the top 20 vital signaling pathways. As shown in Figure 5, many signaling pathways are closely associated with RA, such as the TNF-α signaling pathway, Toll-like receptor signaling pathway, cell apoptosis, IL-17 signaling pathway, C-type lectin receptor signaling pathway, and mitogen-associated protein kinase (MAPK) signaling pathway*.* Additionally, the vital TNF-α signaling pathway is shown in Figure 6.

### Binding Capacity Between the Active Ingredients and TNF-α by Molecular Docking

**FIGURE 7:**
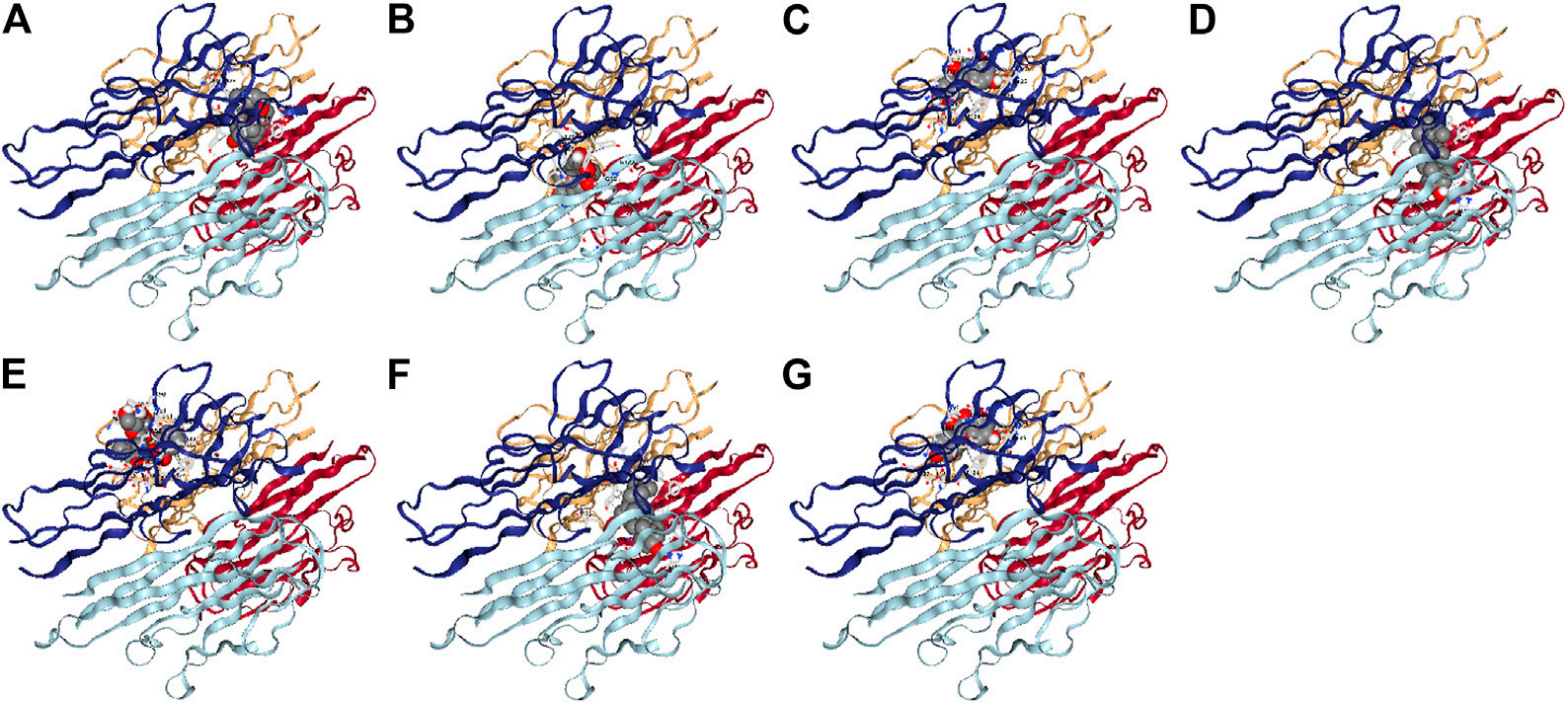
The 3D map of the binding of mairin. **(A)** DPHCD, **(B)** (+)-catechin, **(C)** beta-sitosterol, **(D)** paeoniflorin, **(E)** sitosterol, **(F)** kaempferol, and **(G)** with TNF-α.

Seven active components selected from the WP–RA-potential target gene network bind TNF-α to varying degrees (Table 4). Lower vina scores indicate a stronger and stable interaction between the compound and receptor. The vina scores of mairin, DPHCD, (+)-catechin, beta-sitosterol, paeoniflorin, sitosterol, and kaempferol increased sequentially, indicating that mairin had the strongest and most stable binding affinity toward TNF-α. These results suggest that mairin may be the most appropriate material basis for a natural TNFis. The 3D map of the binding of mairin, DPHCD, (+)-catechin, beta-sitosterol, paeoniflorin, sitosterol, and kaempferol to TNF-α is shown in Figure 7.

**TABLE 4:** Molecular docking parameters and results of seven active ingredients in WP binding with TNF-α.

Molecule ID	Molecule name	Molecule structure	Vina scores	Cavity size	Center^a^			Size^b^		
					*X*	*y*	*z*	*x*	*y*	*z*
MOL000211	Mairin	fx1	−9.4	266	−17	72	36	22	22	22
MOL000359	DPHCD	fx2	−9.0	226	−12	69	17	21	21	21
MOL000492	(+)-catechin	fx3	−8.8	1,258	−6	82	28	21	21	21
MOL001919	Beta- sitosterol	fx4	−8.6	266	−17	72	36	25	25	25
MOL000211	Paeoniflorin	fx5	−8.5	1,258	−6	82	28	22	22	22
MOL001924	Sitosterol	fx6	−8.4	266	−17	72	36	25	25	25
MOL000422	Kaempferol	fx7	−8.1	1,258	−6	82	28	21	21	21

DPHCD:3S,5R,8R,9R,10S,14S)-3,17-dihydroxy-4,4,8,10,14-pentamethyl-2,3,5,6,7,9-hexahydro-1H-cyclopenta[a]phenanthrene-15,16-dione.

a Docking pocket center coordinates.

b The size in the *x*, *y*, and *z* directions of the docking pocket.

## Discussion

RA is a chronic autoimmune disease characterized by inflammatory changes in the synovial tissues, cartilage, and bones of the joints, ultimately leading to disability and death (Scherer et al., 2020). Currently, the incidence of RA is on the rise, which poses a serious threat to health and quality of life (Safiri et al., 2019; Myasoedova et al., 2020). The mechanism of RA is related to multiple factors such as inflammatory immune cells, noninflammatory immune cells, and cytokine/receptor signaling pathways (Wei, 2016). In the present study, 13 active ingredients of WP were found to play significant roles in the treatment of RA and to be associated with a variety of proteins and signaling pathways, suggesting that these active ingredients have a potential research value.

The 13 active ingredients extracted from WP have been widely investigated and confirmed, and have been shown to exert beneficial effects on inflammation and immunity. In an earlier study, Tan et al. (2010) obtained 15 active ingredients from WP by chromatography, including paeoniflorin, albiflorin, beta-sitosterol, and benzoyl paeoniflorin. Similar results were found in other studies (Ren et al., 2009; Fu et al., 2013; Zhu and Fang, 2014; Yan et al., 2020). Other research found that beta-sitosterol significantly inhibited cytokines such as inducible nitric oxide synthase (iNOS), TNF-α, IL-1β, IL-10, and clusters of differentiation 86 (CD86) in M1-polarized macrophages in a concentration-dependent manner in CIA rats (Liu et al., 2019). In addition, beta-sitosterol has a significant therapeutic effect on alleviating arthritic swelling and reducing the level of collagen-specific antibodies (immunoglobulin G (IgG) and IgG1). Furthermore, beta-sitosterol enhances anti-inflammatory activity *via* the suppression of nuclear factor kappa-B (NF-κB) and the activation of the heme oxygenase 1 (NO-1)/ nuclear factor E2-related factor 2 (Nrf2) signaling pathway when it is made into a solid lipid nanoformulation (Zhang et al., 2020). For other compounds studied, evidence showed that (+)-catechin normalizes the gene expression levels of inflammatory cytokines such as COX-2, TNF-α, IL-1β, IL-6, and NF-κB (Yimam et al., 2013; Fechtner et al., 2017). Similarly, numerous studies have confirmed the anti-inflammatory and immunomodulatory effects of paeoniflorin in a variety of animal models, and synovial and inflammatory cells from RA patients (Zhang et al., 2019; Zhang and Wei, 2020). Overall, seven active ingredient compounds have different degrees of therapeutic effects on RA, involving a variety of cytokines and signaling pathways.

As previously mentioned, TGP, an extract of WP, contains the main components of WP and has excellent anti-inflammatory and immunomodulatory effects on RA. Clinical trials have found that TGP is effective in the clinical treatment of RA patients, significantly improving clinical indicators such as arthralgia, joint swelling, the joint swelling index, joint induration, stiffness, and grip strength, and reducing the erythrocyte sedimentation rate, C-reactive protein, and rheumatoid factor (Wang et al., 2001; Min et al., 2005). Studies performed in RA animal models showed that TGP significantly reduced the swelling of the arthritic foot and paw in rats with AA and CIA, modulated abnormal immune function in AA and CIA rats, reduced serum anti–type II collagen antibodies in CIA, and inhibited the secretion of IL-1, TNF-α, and other inflammatory cytokines by abdominal macrophages. TGP was also shown to decrease serum IL-17 levels and regulate the balance of mesenteric T helper one lymphocytes (Th1) and Th2 cells in AA. It inhibits the proliferation of synovial cells and the production of cytokines such as IL-1, TNF-α, and prostaglandin E2 (Jia et al., 2014; Zhang and Wei, 2020). These findings suggest that multiple active ingredients in WP work together in a synergistic manner to treat RA.

As seen in the WP–RA-potential target gene network (Figure 3), many target genes can be regulated by multiple compounds in RA. These genes include but are not limited to PGR, PTGS1, NR3C2, TNFSF15, JUN, and CASP3. Conversely, IL-6, ADRB2, NOS2, catalase (CAT), and CYP3A4 were the only target genes for specific compounds in the network. These results suggest that WP therapy for RA has multicomponent, multi-targeted biological attributes. Additionally, the PPI results suggest that the 49 target proteins are not independent of each other, but are linked and interact (Zeng et al., 2019). These results also indicate that WP can be involved in the alleviation and the treatment of RA through the regulation of multiple proteins. As shown in Figure 2, IL-6, AKT1, PTGS2, and JUN were the vital target genes in the PPI network.

Analysis of GO and KEGG enrichment results for the 49 target genes suggested that 79 biological functions and 50 signaling pathways are directly involved in the occurrence and development of RA, suggesting that these gene biological functions and signaling pathways may be the mechanism by which WP can treat RA. The pathways with the best correlation are selected here for a discussion on the mechanism of the WP treatment for RA. TNF-α is not only a pro-inflammatory cytokine but also initiates various signaling pathways in the immune system in RA (Mateen et al., 2016). Inhibition of TNF-α expression and TNF-α antibody therapy can effectively reduce arthritis and synovitis symptoms in RA patients (Matsuno et al., 2002). Different doses of TGP and paeoniflorin reduced the expression and serum concentration levels of TNF-α, which was secreted by peritoneal macrophages and fibroblast-like synoviocytes (FLSs) in AA and CIA rats (Xu et al., 2007; Zhang et al., 2008b; Wang et al., 2015). The Toll-like receptor signaling pathway contributes to many autoimmune diseases such as RA (Thwaites et al., 2014). Toll-like receptors exert significant effects on immune responses and are involved in the proliferation, survival, and apoptosis of inflammatory cells (Kutikhin, 2011). It has been reported that endogenous activation of the Toll-like receptor signaling pathway can exacerbate synovial inflammation in RA patients (Monaco et al., 2011). The study of the Toll-like receptor signaling pathway helps to reveal the pathogenesis of RA and reduces abnormal inflammation, providing potential targets. Autophagy is an important homeostatic process by which cells breakdown their components. Recent studies have revealed the critical role of apoptotic and autophagic pathways in immunity and inflammation, which balance the beneficial and harmful effects of immunity and inflammation, and therefore prevent the development of RA (Levine et al., 2011; Nygaard et al., 2018). Kim et al. (2017) found that IL-17 induced mitochondrial dysfunction and autophagosome formation in RA FLSs, suggesting resistance to apoptosis by IL-17. In addition, IL-17–induced autophagy-related antiapoptotic effects are restored by inhibition of autophagy, suggesting a relationship between mitochondrial dysfunction and cell survival in RA FLSs. IL-17 and Th17 cells play an important role in the development of RA. Research from animal experimental models suggests a role for IL-17 in pannus growth, structural destruction of rheumatoid joints through NF-κB receptor activator-dependent osteoclast formation, and synovial damage (Pickens et al., 2010; Ito et al., 2011). The MAPK signaling pathway is widely studied for its involvement in regulating the expression of multiple genes associated with inflammation in RA (Li et al., 2017; Zou et al., 2019). The MAPK signaling pathway includes extracellular signal-regulated kinase (ERK), Jun N-terminal kinase (JNK), and p38, which are closely associated with T-cell activation, the proliferation of FLSs, the production of inflammatory cytokines, and the induction of joint damage (Schett et al., 2000; Pearson et al., 2001). It was found that WP and its active ingredients can inhibit the phosphorylation levels of ERK, JNK, and p38 in FLSs and synovial tissues of CIA and AA rats, which provides a potential mechanism for the anti-inflammatory and immunomodulatory effects of WP (Zheng and Wei, 2005; Zhang et al., 2008a).

In this research, it was found that mairin with better OB and DL properties was screened in WP, as it had the optimal molecular binding capacity to TNF-α.The results from a recent study demonstrated that mairin decreased the level of TNF-α in RA-FLSs (Wang and Zhao, 2018). Furthermore, mairin inhibits the activation of the protein kinase B/ NF-κB pathway in TNF-α–exposed RAFLSs, thereby alleviating RA-FLS proliferation, migration, and the inflammatory response. AA and CIA model experiments found that mairin inhibits paw swelling; arthritis index; joint pathology such as synovial tissue hyperplasia; cartilage destruction; vasospasm; inflammatory cytokines such as TNF-α, IL-1β, IL-6, IL-8, and IL-17A; and RA target proteins such as endothelial growth factor and transforming growth factor β (Mathew and Rajagoapl, 2017; Wang and Zhao, 2018; Huimin et al., 2019; Kun et al., 2020). These findings indicated that mairin has excellent therapeutic effects on RA and inhibits TNF-α. This could then validate our findings. However, the limitation of this study is that the interaction between the active ingredients was not considered. In addition, the absorption of compounds in humans is not limited to OB. Further studies need to be completed for experimental validation. Taking these findings into consideration provides a theoretical basis for further development of natural TNFis and the development of new anti-inflammatory immune drugs using WP as the base substance.

## Conclusion

Taken together, WP has obvious advantages in the treatment of RA, which is consistent with previous studies. The biological functions and signaling pathways of the WP active ingredients on RA target genes were investigated by the network pharmacology approach. Meanwhile, mairin, with optimal molecular binding to TNF-α, was obtained by the molecular binding assay and can be researched as the most appropriate natural TNFis and as a lead compound for further structural modification and development. These findings will further reveal the molecular biological mechanism of WP in the treatment of RA and provide a theoretical basis for the clinical treatment of RA.

## Data Availability

The datasets presented in this study can be found in online repositories. The names of the repository/repositories and accession number(s) can be found in the article/Supplementary Material.
