# Supplementary material for: Identification of Tumor Necrosis Factor-Alpha (TNF-α) Inhibitor in Rheumatoid Arthritis Using Network Pharmacology and Molecular Docking
Source: Front Pharmacol. 2021 May 21;12:690118. doi: 10.3389/fphar.2021.690118 (PMC8175775; doi:10.3389/fphar.2021.690118)
Supplement: Supplementary file 1 [file DataSheet1.DOCX]

***Supplementary Material***

**1 Supplementary Data**

**1.1 Supplementary Table**

**Supplementary Table 1** The 95 target genes of WP active ingredients

| **NO** | **Molecule ID** | **Molecule Name** | **Target** | **Symbol** |
| --- | --- | --- | --- | --- |
|  | MOL001918 | Paeoniflorgenone | Gamma-aminobutyric acid receptor subunit alpha-1 | GABRA1 |
|  | MOL001919 | DPHCD | Progesterone receptor | PGR |
|  | MOL001919 | DPHCD | Mineralocorticoid receptor | NR3C2 |
|  | MOL001924 | Paeoniflorin | Tumor necrosis factor | TNFSF15 |
|  | MOL001924 | Paeoniflorin | Interleukin-6 | IL6 |
|  | MOL001924 | Paeoniflorin | Monocyte differentiation antigen CD14 | CD14 |
|  | MOL001924 | Paeoniflorin | Lipopolysaccharide-binding protein | LBP |
|  | MOL000211 | Mairin | Progesterone receptor | PGR |
|  | MOL000358 | Beta-sitosterol | Progesterone receptor | PGR |
|  | MOL000358 | Beta-sitosterol | Nuclear receptor coactivator 2 | NCOA2 |
|  | MOL000358 | Beta-sitosterol | Prostaglandin G/H synthase 1 | PTGS1 |
|  | MOL000358 | Beta-sitosterol | Prostaglandin G/H synthase 2 | PTGS2 |
|  | MOL000358 | Beta-sitosterol | Potassium voltage-gated channel subfamily H member 2 | KCNH2 |
|  | MOL000358 | Beta-sitosterol | Muscarinic acetylcholine receptor M3 | CHRM3 |
|  | MOL000358 | Beta-sitosterol | Muscarinic acetylcholine receptor M1 | CHRM1 |
|  | MOL000358 | Beta-sitosterol | Sodium channel protein type 5 subunit alpha | SCN5A |
|  | MOL000358 | Beta-sitosterol | Muscarinic acetylcholine receptor M4 | CHRM4 |
|  | MOL000358 | Beta-sitosterol | Alpha-1A adrenergic receptor | ADRA1A |
|  | MOL000358 | Beta-sitosterol | Muscarinic acetylcholine receptor M2 | CHRM2 |
|  | MOL000358 | Beta-sitosterol | Alpha-1B adrenergic receptor | ADRA1B |
|  | MOL000358 | Beta-sitosterol | Beta-2 adrenergic receptor | ADRB2 |
|  | MOL000358 | Beta-sitosterol | Neuronal acetylcholine receptor subunit alpha-2 | CHRNA2 |
|  | MOL000358 | Beta-sitosterol | Sodium-dependent serotonin transporter | SLC6A4 |
|  | MOL000358 | Beta-sitosterol | Mu-type opioid receptor | OPRM1 |
|  | MOL000358 | Beta-sitosterol | Gamma-aminobutyric acid receptor subunit alpha-1 | GABRA1 |
|  | MOL000358 | Beta-sitosterol | Apoptosis regulator Bcl-2 | BCL2 |
|  | MOL000358 | Beta-sitosterol | Apoptosis regulator BAX | BAX |
|  | MOL000358 | Beta-sitosterol | Caspase-9 | CASP9 |
|  | MOL000358 | Beta-sitosterol | Transcription factor AP-1 | JUN |
|  | MOL000358 | Beta-sitosterol | Caspase-3 | CASP3 |
|  | MOL000358 | Beta-sitosterol | Caspase-8 | CASP8 |
|  | MOL000358 | Beta-sitosterol | Protein kinase C alpha type | PRKCA |
|  | MOL000358 | Beta-sitosterol | Serum paraoxonase/arylesterase 1 | PON1 |
|  | MOL000358 | Beta-sitosterol | Microtubule-associated protein 2 | MAP2 |
|  | MOL000359 | Sitosterol | Progesterone receptor | PGR |
|  | MOL000359 | Sitosterol | Nuclear receptor coactivator 2 | NCOA2 |
|  | MOL000359 | Sitosterol | Mineralocorticoid receptor | NR3C2 |
|  | MOL000422 | Kaempferol | Nitric oxide synthase, inducible | NOS2 |
|  | MOL000422 | Kaempferol | Prostaglandin G/H synthase 1 | PTGS1 |
|  | MOL000422 | Kaempferol | Androgen receptor | AR |
|  | MOL000422 | Kaempferol | Peroxisome proliferator activated receptor gamma | PPARG |
|  | MOL000422 | Kaempferol | Prostaglandin G/H synthase 2 | PTGS2 |
|  | MOL000422 | Kaempferol | Nuclear receptor coactivator 2 | NCOA2 |
|  | MOL000422 | Kaempferol | Trypsin-1 | PRSS1 |
|  | MOL000422 | Kaempferol | Progesterone receptor | PGR |
|  | MOL000422 | Kaempferol | Muscarinic acetylcholine receptor M1 | CHRM1 |
|  | MOL000422 | Kaempferol | Acetylcholinesterase | ACHE |
|  | MOL000422 | Kaempferol | Sodium-dependent noradrenaline transporter | SLC6A2 |
|  | MOL000422 | Kaempferol | Muscarinic acetylcholine receptor M2 | CHRM2 |
|  | MOL000422 | Kaempferol | Alpha-1B adrenergic receptor | ADRA1B |
|  | MOL000422 | Kaempferol | Gamma-aminobutyric acid receptor subunit alpha-1 | GABRA1 |
|  | MOL000422 | Kaempferol | Coagulation factor VII | F7 |
|  | MOL000422 | Kaempferol | Transcription factor p65 | RELA |
|  | MOL000422 | Kaempferol | Inhibitor of nuclear factor kappa-B kinase subunit beta | IKBKB |
|  | MOL000422 | Kaempferol | RAC-alpha serine/threonine-protein kinase | AKT1 |
|  | MOL000422 | Kaempferol | Apoptosis regulator Bcl-2 | BCL2 |
|  | MOL000422 | Kaempferol | Apoptosis regulator BAX | BAX |
|  | MOL000422 | Kaempferol | Tumor necrosis factor | TNFSF15 |
|  | MOL000422 | Kaempferol | Transcription factor AP-1 | JUN |
|  | MOL000422 | Kaempferol | Activator of 90 kDa heat shock protein ATPase homolog 1 | AHSA1 |
|  | MOL000422 | Kaempferol | Caspase-3 | CASP3 |
|  | MOL000422 | Kaempferol | Mitogen-activated protein kinase 8 | MAPK8 |
|  | MOL000422 | Kaempferol | Interstitial collagenase | MMP1 |
|  | MOL000422 | Kaempferol | Signal transducer and activator of transcription 1-alpha/beta | STAT1 |
|  | MOL000422 | Kaempferol | Peroxisome proliferator-activated receptor gamma | PPARG |
|  | MOL000422 | Kaempferol | Heme oxygenase 1 | HMOX1 |
|  | MOL000422 | Kaempferol | Cytochrome P450 3A4 | CYP3A4 |
|  | MOL000422 | Kaempferol | Cytochrome P450 1A2 | CYP1A2 |
|  | MOL000422 | Kaempferol | Cytochrome P450 1A1 | CYP1A1 |
|  | MOL000422 | Kaempferol | Intercellular adhesion molecule 1 | ICAM1 |
|  | MOL000422 | Kaempferol | E-selectin | SELE |
|  | MOL000422 | Kaempferol | Vascular cell adhesion protein 1 | VCAM1 |
|  | MOL000422 | Kaempferol | Nuclear receptor subfamily 1 group I member 2 | NR1I2 |
|  | MOL000422 | Kaempferol | Cytochrome P450 1B1 | CYP1B1 |
|  | MOL000422 | Kaempferol | Arachidonate 5-lipoxygenase | ALOX5 |
|  | MOL000422 | Kaempferol | Hyaluronan synthase 2 | HAS2 |
|  | MOL000422 | Kaempferol | Glutathione S-transferase P | GSTP1 |
|  | MOL000422 | Kaempferol | Aryl hydrocarbon receptor | AHR |
|  | MOL000422 | Kkaempferol | 26S proteasome non-ATPase regulatory subunit 3 | PSMD3 |
|  | MOL000422 | Kaempferol | Solute carrier family 2, facilitated glucose transporter member 4 | SLC2A4 |
|  | MOL000422 | Kaempferol | Nuclear receptor subfamily 1 group I member 3 | NR1I3 |
|  | MOL000422 | Kaempferol | Insulin receptor | INSR |
|  | MOL000422 | Kaempferol | Type I iodothyronine deiodinase | DIO1 |
|  | MOL000422 | Kaempferol | Serine/threonine-protein phosphatase 2B catalytic subunit alpha isoform | PPP3CA |
|  | MOL000422 | Kaempferol | Glutathione S-transferase Mu 1 | GSTM1 |
|  | MOL000422 | Kaempferol | Glutathione S-transferase Mu 2 | GSTM2 |
|  | MOL000422 | Kaempferol | Aldo-keto reductase family 1 member C3 | AKR1C3 |
|  | MOL000422 | Kaempferol | Antileukoproteinase | SLPI |
|  | MOL000492 | (+)-catechin | Prostaglandin G/H synthase 1 | PTGS1 |
|  | MOL000492 | (+)-catechin | Estrogen receptor | ESR1 |
|  | MOL000492 | (+)-catechin | Prostaglandin G/H synthase 2 | PTGS2 |
|  | MOL000492 | (+)-catechin | Nuclear receptor coactivator 2 | NCOA2 |
|  | MOL000492 | (+)-catechin | Retinoic acid receptor RXR-alpha | RXRA |
|  | MOL000492 | (+)-catechin | Catalase | CAT |
|  | MOL000492 | (+)-catechin | Hyaluronan synthase 2 | HAS2 |

Note:DPHCD:(3S,5R,8R,9R,10S,14S)-3,17-dihydroxy-4,4,8,10,14-pentamethyl-2,3,5,6,7,9-hexahydro-1H-cyclopenta[a]phenanthrene-15,16-dione

**1.2 Supplementary Figures**


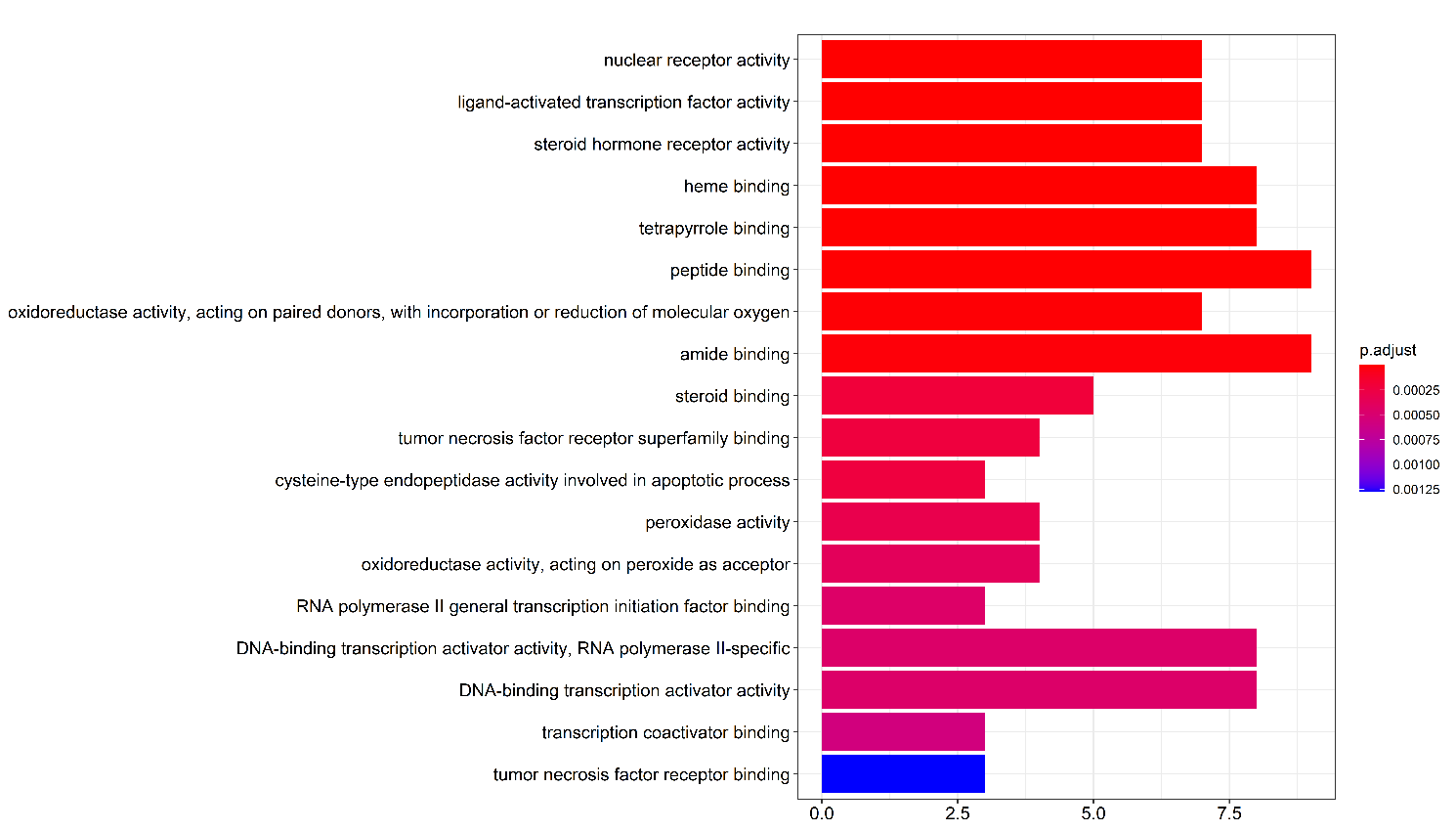


**Supplementary Figure 1.** The top 18 remarkably enriched GO analysis for biological function of potential target genes of WP in RA.

**
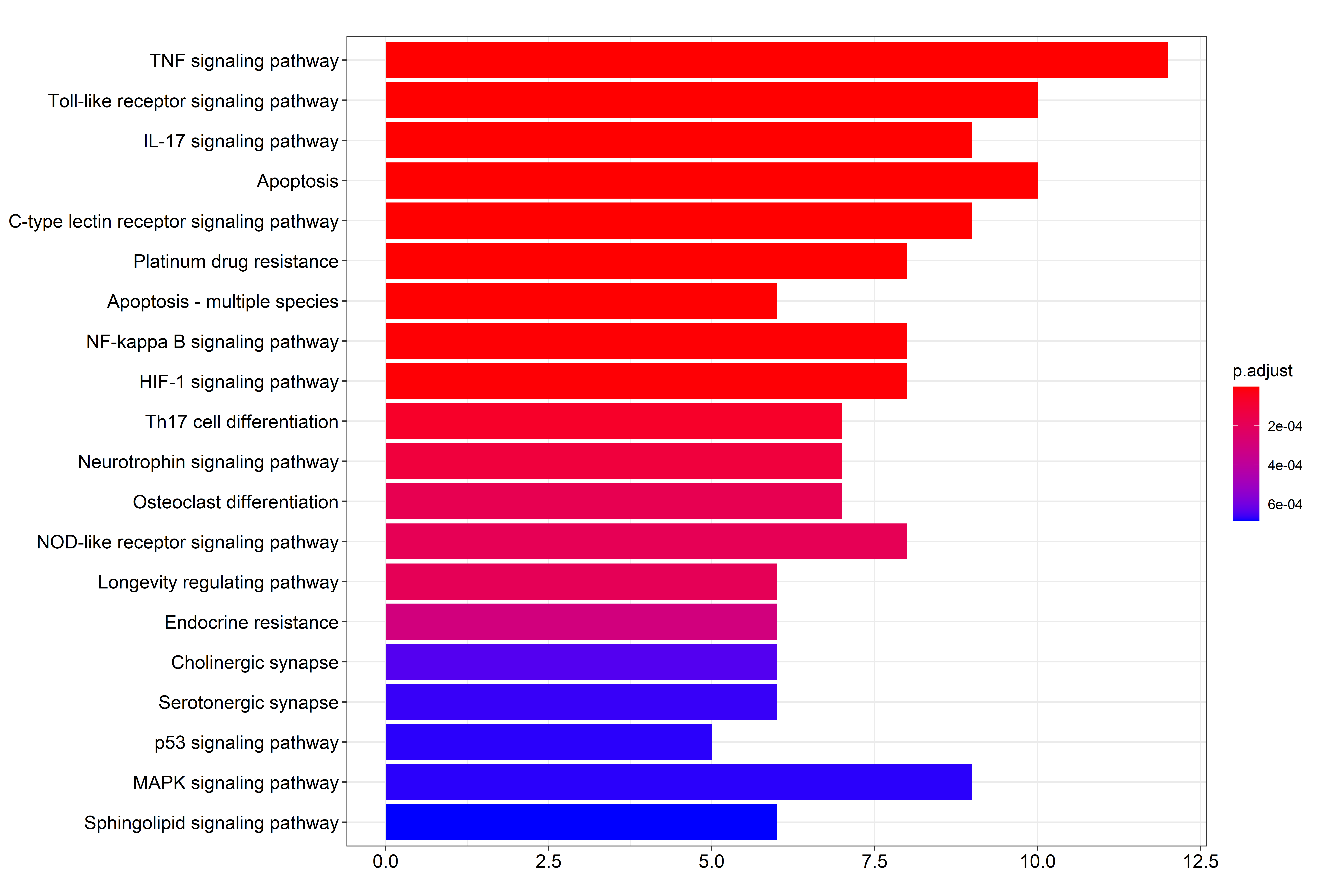
****Supplementary Figure 2.**  The top 20 remarkably enriched KEGG analysis for signaling pathway of potential target genes of WP in RA.
